# Traveling Letter, No. VI

**Published:** 1844-10

**Authors:** 


### TRAVELING LETTERS FROM THE SENIOR EDITOR.

NO. VI.

St. Louis, August 31st, 1844.

*To my Colleagues:*

Gentlemen:—Although I wrote to you only three days ago, I am, as you see, about to write again. Being in St. Louis, where my notes of travel in the south are deposited, and on the eve of my departure for the Upper Mississippi and Lake Michigan, the time seems favorable for giving you a rambling notice of a disease which has prevailed in several western and frontier localities, and excited a good deal of interest among the people. I allude to

### *Black-Tongue.*

I begin by telling you, that I have not seen a single case of it, and that the promised circumstantial narratives of the two gentlemen in the south, who had seen more of it than any others whom I had met, have not yet been received. Under the expectation of a written narrative from each of them, I recorded no part of what they told me, and have not, therefore, as ample a stock of materials for this letter, as I hoped, by deferring it, would come into my possession.

The places farthest south, where I heard of its prevailing, were Grand Gulf and Port Gibson in Mississippi in latitude nearly 32°; the town farthest north, Boonville in Missouri, which is about the 39°th. Thus it has extended through 7 degrees of latitude, and is evidently not connected exclusively with the south or the north. Nor is it either civic or rural, but both, and affects whites and blacks—the last, perhaps, more frequently and fatally than the former. Finally, it occurs in both summer and winter, as it was prevailing in the country near Vicksburg, when I was there in July, and began to prevail in Boonville in the month of December. As to the mode of its dissemination I may say, that, as in the case of scarlatina, there are *apparently* well authenticated facts showing it to be contagious, and also, to arise independently of contagion. But what *is* the malady, which has received from the people the frightful appellation, with which I have signalized my sheet? Why, according to all the physicians who have had opportunities for observation, it is not exactly *the*, but *an Erysipelas;* that is, a St. Anthony's fire, complicated with angina, and sometimes with an efflorescence, which some physicians have called scarlatina roseola, for in fact, in different places and persons, it seems to have displayed the characteristics of each. It does not appear that the local and constitutional affections have preserved a constant order of occurrence, for in some cases the former, and in others the latter, have appeared first. This is true, however, of the angina only, for I believe that the fever has always preceded the cutaneous inflammation, whether erysipelatous or papular. In some neighborhoods, while a few severe cases combining all these elements have occurred, and even proved fatal, a much larger number have run their course as mild anginas, not even confining the patients to their houses. In certain cases there was no quinsy, and the gastrodynia and vomiting clearly indicated, that the stomach instead of the throat, was the seat of inflammation; which was further evinced by the early death of the patient.

The erysipelas generally showed itself on the face. Sometimes spreading from the throat through the nostrils, but oftener appearing first about the inner canthus of the eye, having perhaps ascended the nasal duct. Diffusion, however, by the continuous sympathy of John Hunter, was not necessary to the cutaneous inflammation, for occasionally it appeared first on the cheek, the neck, the shoulder, the side, the extremities, and even on the scrotum. Still the face and head were affected in the majority of cases, and the inflammation for the most part continued spreading, till the whole were involved. The eyelids were generally so swollen as to be closed for many days; nevertheless it does not appear that the conjunctiva was often inflamed, though in one case an eye was lost. When the inflammation of the scalp was severe, depilation generally followed, and in all cases there was a copious desquamation of the cuticle, whatever part might be affected. The inflammation was so strongly phlegmonous, that extensive suppurations in the subjacent cellular membrane were ordinary events. It also manifested a decided gangrenous tendency, to which the death of the patient was often referable. As the erysipelas appeared, the angina generally ceased. In one instance the former appeared first, and the latter followed. It was in a physician of Port Gibson, and as his case illustrates several phenomena of the disease, and he once lived in Louisville, where he was generally known, I will give you a short history of it-

Dr. Morehead was called from Port Gibson to the neighboring town of Grand Gulf, in the month of April last, to attend on a friend affected with the disease, which was then epidemic in that place. The attack was severe and his patient died. He was constant in his attendance, and performed the functions of nurse, as well as physician. One day, in making an application to the throat, the secretions from it came in contact with an abraded spot on one of the fingers of his left hand. Soon afterwards the parts showed signs of inflammation, and the lymphatic vessels of the axilla became sore and red. Fever supervened, and an erysipelatous inflammation appeared on his face, which penetrated his nostrils, and extended to his throat. At the same time, an erysipelato-phlegmonous inflammation attacked his side, which suppurated. Finally, gangrene appeared in both the face and the side, and in a fortnight he died. Still further to illustrate the symptomatology of this affection, I may as well give you the leading symptoms of another case, especially as it is intimately connected with the last,

Dr. Harper, of Port Gibson, attended Dr. Morehead throughout his illness. In a few days afterwards having taken a shower bath at night, he was seized next morning with fever and a severe affection of the larynx, attended with hoarseness and aphonia, but no cough. The inflammation became submucous, and produced such straining on the laryngeal passages, that he could scarcely breathe. His deglutition was difficult, and he experienced considerable coma and delirium. His tongue appeared to be a little swollen, and was covered with fur, at first white, and afterwards very dark, rendered so perhaps by the solution of nitrate of silver, which was applied to his throat- At length the inflammation traveled into his posterior nares, and finally reached his nostrils. He had no sneezing, but the mucous membrane was so swollen, that he breathed through his mouth and the secretion of mucus was suspended. From the nostrils the inflammation advanced upon his face, at the same time ceasing in the larynx and pharynx. On assailing the skin it first fell on the left side and then advanced across the nose to the right, until the whole face was involved. No suppuration nor gangrene supervened, and he recovered.

In some instances the tongue was so swollen and inflamed as to make glossitis the prominent part of the disease. I have heard of another patient, besides Dr. Morehead, in whom the inflammation began in a small injury of one of the fingers, and extended to the body; showing that *his* disease might not have arisen from inoculation.

At some places, the rash occurred in a majority of cases, while at other places, it passed unobserved or was entirely wanting. In its least degree it presented a few patches only of redness or minute pimples—in its highest, the body was pretty extensively covered. By several physicians, it was identified in its visible characters with scarlatina; but others thought that it bore a greater resemblance, as I have intimated, to roseola. In all its grades and varieties it was followed by furfuraceous desquamation of the cuticle; and I was told of one case, in which the rash had at least not been observed, and yet there was universal desquamation, at the time the cuticle from the inflamed surface peeled off.

Vesicles or blebs, sometimes appeared in the midst of the rash, at others replaced it. In a few instances they had inflamed bases, were indented and resembled the variolous eruption. But their number in these cases was not great.

Slight œdema of the ankles, and frequently of the face and hands, very generally attended the convalescence of the patient.

Before I proceed to speak of the treatment of this malady, let us look for a moment at its nosological elements. In doing this, it seems to me to be a compound of erysipelas and scarlatina. The latter disease had been for many years prevailing, here and there, in the Valley of the Mississippi, and the former, especially in hospitals, has often shown an epidemic tendency—was actually epidemic in our hospital, last winter and spring. Now, if we suppose this tendency to be augmented, in places where the cause of scarlatina prevailed, we may admit the production of a mongrel compound affection. But to this admission, there is this serious objection, that scarlatina in general affects the same person but once, while the disease under consideration attacks so many adults, that we may *suppose* there were among them a number, who had experienced scarlet fever. Nevertheless as numerous children grow up without having had scarlatina it *may* be, that the adult subjects of this disease were of that class. The symptoms of the new visiter certainly direct the mind to the conclusion which I have presented; but I do not consider it as established. The œdema, however, which followed the disease, seems, in an especial manner, to indicate the presence of the cause of scarlatina. Again, the late epidemic has obeyed precisely the laws of propagation of scarlatina. Finally, the latter, within the past year, has, in several localities, blended itself with measles, of which curious examples were related to me by Dr. Ames, of Montgomery, and Dr. Fearn, of Mobile, when I was in Alabama: now if it can modify that disease, I know no reason why it should not modify erysipelas. Were I to *dwell* on this proposition, I might not establish it, and if I did, the utility of the demonstration would not, perhaps, be very great; I shall therefore dismiss it, and proceed to speak of the treatment.

Most of the gentlemen with whom I have conversed employed the lancet in the early stages of the disease, with advantage. The blood was buffed. Some patients were bled as much as four times; the majority not more than once; many not at all; and in general bleeding after the third day of the fever was found prejudicial, by promoting a typhoid condition, or, in the south, a state of collapse. Dr. Harper, whose case I have mentioned, was bled three or four times in one day, to relieve him from œdematous laryngitis. In many cases, not attended with retrocession of the inflammation of the skin, the brain suffered severely, as was evidenced by stupor or delirium, all of which called for a liberal use of the lancet. In others the lungs were deeply involved, and the patient, along with dyspnœa and sense of suffocation, complained of a burning sensation throughout the chest, a condition which likewise demanded copious venesection. When the stomach and bowels became implicated, except when the strength of the patient was rapidly exhausted by profuse diarrhœa, bloodletting was also practised with advantage. Cupping was resorted to by some physicians. When performed on the nucha it relieved the throat and brain, and often gave relief to the gastric symptoms, when the blood was drawn from the pit of the stomach.

Emetics were not much used, but when the stomach was not affected they were sometimes administered, and contributed to remove the quinsy. Nauseants were employed by some with advantage.

Cathartics were very generally employed, and found beneficial when the stomach was not irritable. Calomel, followed by senna or oil, was commonly used. But they were not given in large doses, and *copious* purging was not on the whole found beneficial.

Opium was requisite, when profuse diarrhœa occurred, and, after venesection and purging, did good, except when the brain was affected. This was so often the case, however, that narcotics had to be prescribed with circumspection.

After the first few days, the powers of the system in many cases, seemed to be exhausted, and diffusible stimuli, such as ammonia, camphor, and wine, with nutrients and tonics, including the sulphate of quinine, were demanded, and produced a good effect. In extensive suppurations, and under a tendency to gangrene, this class of remedies was particularly required, and then opium was not only safe but decidedly useful.

A great variety of topical applications were employed. To the throat, an infusion of rose leaves acidulated with muriatic acid, infusion of capsicum, a solution of corrosive sublimate, tincture of myrrh; a mixture of oil of turpentine, gum arabic and honey, a solution of nitrate of silver, and the same medicine in a solid form, with many other applications of a like kind, were resorted to by different physicians. It will be observed by you, that all were of a stimulating quality, and, perhaps, they were equally beneficial. A blister to the throat or the nape of the neck, or the application of croton oil externally, contributed by counterirritation, to the same effect with those to the inside.

To the inflamed skin an equal variety of medicaments have been applied, but so much diversity of opinion as to their efficacy exists, that one is led to believe none of them to have been very serviceable. Blisters were very generally employed, but while some physicians found them successful, others were disappointed. When they covered the inflamed surfaces as well as the surrounding parts, they were found by some practitioners more beneficial than when confined to the latter. Mercurial ointment of the ordinary strength, or diluted with lard, was a common application, which in the practice of some did good—in that of others harm: at least such was the estimates of its effects. In several cases covering the skin with olive oil and flour, then applying bats of cotton, and, finally, a roller bandage, when the part admitted of such an application, was found beneficial. Solutions of nitrate of silver and corrosive sublimate were applied, with variable results, by different physicians; but I could not learn, that the latter was, in any case, of the strength of ʒij to the ℥j, of water, as recommended in our journal, by Drs. Tripler and Pitcher, for ordinary erysipelas. Some physicians found diluted tincture of camphor good; others used a solution of sugar of lead, or of muriate of ammonia; and others placed their reliance on mucilages of elm bark and flaxseed.

I might extend this catalogue of local remedies, but utility does not demand it; and I will close by mentioning the names of the gentlemen from whom, chiefly, I collected the facts which I have here embodied:—Drs. Abbay, Harper, Peck, Todd, and Maury, Port Gibson; Dr. Gunby, Grand Gulf; Dr. Langley, Jackson; Dr. Dorsey, Yazoo City; Dr. Hicks, Vicksburg, all of the State of Mississippi. Also, Drs. Hart, Thomas, Stockton, Shortridge, and Redwood, of Boonville, and Professor Hall, of St. Louis, in Missouri. I have thought it unnecessary in a letter to go into the formality of connecting the names of these gentlemen with particular facts.

Respectfully yours,

